# Crosstalk between Oxidative Stress and Inflammatory Liver Injury in the Pathogenesis of Alcoholic Liver Disease

**DOI:** 10.3390/ijms23020774

**Published:** 2022-01-11

**Authors:** Yoon Mee Yang, Ye Eun Cho, Seonghwan Hwang

**Affiliations:** 1 Department of Pharmacy, Kangwon National University, Chuncheon 24341, Korea; yym@kangwon.ac.kr; 2 KNU Researcher Training Program for Developing Anti-Viral Innovative Drugs, Kangwon National University, Chuncheon 24341, Korea; 3 Department of Manufacturing Pharmacy, College of Pharmacy, Pusan National University, Busan 46241, Korea; eloss98@pusan.ac.kr; 4 Research Institute for Drug Development, Pusan National University, Busan 46241, Korea

**Keywords:** alcoholic liver disease, oxidative stress, inflammatory liver injury, fatty liver, alcoholic steatohepatitis, cirrhosis

## Abstract

Alcoholic liver disease (ALD) is characterized by the injury, inflammation, and scarring in the liver owing to excessive alcohol consumption. Currently, ALD is a leading cause for liver transplantation. Therefore, extensive studies (in vitro, in experimental ALD models and in humans) are needed to elucidate pathological features and pathogenic mechanisms underlying ALD. Notably, oxidative changes in the liver have been recognized as a signature trait of ALD. Progression of ALD is linked to the generation of highly reactive free radicals by reactions involving ethanol and its metabolites. Furthermore, hepatic oxidative stress promotes tissue injury and, in turn, stimulates inflammatory responses in the liver, forming a pathological loop that promotes the progression of ALD. Accordingly, accumulating further knowledge on the relationship between oxidative stress and inflammation may help establish a viable therapeutic approach for treating ALD.

## 1. Introduction

Excessive and chronic alcohol intake can cause numerous problems affecting various physiological systems, including the immune, nervous, cardiovascular, and digestive systems [1,2,3,4,5]. The hepatic manifestation of heavy alcohol consumption is referred to as alcoholic liver disease (ALD), which encompasses a wide spectrum of disorders including fatty liver, alcoholic steatohepatitis (ASH), alcoholic hepatitis (AH), cirrhosis, and hepatocellular carcinoma [6,7,8,9,10]. Fatty liver is relatively benign and represents the initial stage in the ALD spectrum, marked by triglyceride accumulation in the liver. In some individuals, alcoholic fatty liver progresses to ASH, which is characterized by the presence of hepatocyte injury, hepatocyte ballooning, and inflammation [11]. Chronic injury, inflammation, and activation of the liver regeneration machinery, which are features of ASH, may result in the replacement of the hepatic parenchyma with fibrotic tissues, eventually causing liver failure and cirrhosis [12]. Apart from the chronic, subclinical nature of ASH progression, acute and overt syndromes observed in patients with ALD are referred to as AH, known to present a poor prognosis [13].

ALD has become one of the leading causes of end-stage liver disease, and necessitates liver transplantation, while the contribution of viral infections has gradually waned [14,15]. In the United States, recent studies have reported that approximately 40% of cirrhosis-related deaths can be attributed to ALD, and the three-month mortality of severe AH is approximately 50%, indicating that ALD may be fatal without active therapeutic intervention [16,17]. However, therapeutic options for ALD remain limited.

Molecular mechanisms underlying the principal features of ALD progression, including liver injury, inflammation, and fibrosis, have been extensively investigated as potential therapeutic targets for ALD [18]. Numerous reports have demonstrated that the pathogenesis of ALD is often accompanied by oxidative stress and inflammatory injury [19,20]. This review summarizes recent advances in our understanding of the pathogenic roles and interplay between oxidative stress and inflammation during ALD development. In addition, we discuss therapeutic approaches that target oxidative stress and inflammation in ALD.

## 2. Oxidative Stress-Related Pathogenic Mechanisms of ALD

ALD pathogenesis involves various processes, including fat accumulation, organelle stress and hepatocyte death, immune cell infiltration and activation, and fibrogenesis stimulated by hepatic stellate cells [19,21,22,23,24]. As stated above, these processes are reportedly stimulated by and/or enhance oxidative stress. Early studies have revealed that ethanol metabolism via alcohol dehydrogenase (ADH) and microsomal cytochrome P450 (CYP) enzymes produces acetaldehyde and reactive oxygen species (ROS) and depletes glutathione levels [25,26,27,28,29,30]. These findings and other reports have highlighted the importance of oxidative stress in the pathogenesis of ALD.

**Figure 1:**
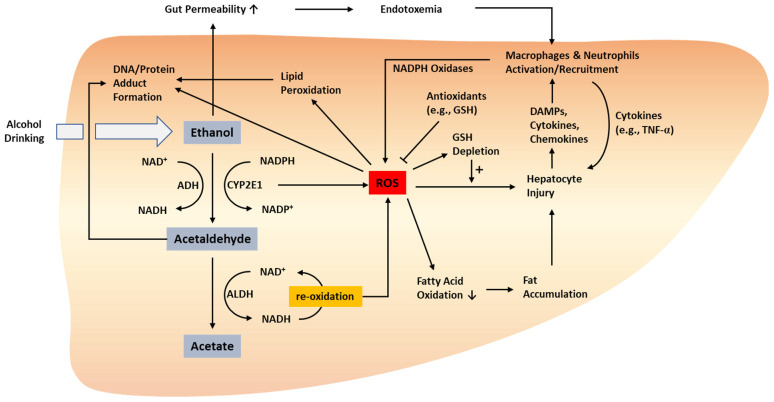
Oxidative stress-related pathogenesis of ALD. ROS can be produced by the metabolism of ethanol to acetaldehyde and acetate as well as the related processes that involve the conversion between NAD^+^/NADP^+^ and NADH/NADPH. ROS produced via these processes stimulate hepatocyte injury directly or via enhanced fat accumulation. Injured hepatocytes release DAMPs, cytokines, and chemokines, which activate and recruit innate immune cells such as macrophages and neutrophils. Activated macrophages and neutrophils can also produce ROS via NADPH oxidase. Protein and DNA adducts formed by acetaldehyde and ROS may facilitate liver injury, inflammation, and carcinogenesis. ADH, alcohol dehydrogenase; ALD, alcoholic liver disease; ALDH, aldehyde dehydrogenase; DAMP, damage-associated molecular pattern; GSH, glutathione; ROS, reactive oxygen species; TNF-α, tumor necrosis factor-alpha. ↑, increased; ↓, decreased.

The oxidation of ethanol to acetaldehyde and acetate utilizes NAD^+^ as a cofactor and produces NADH, thereby reducing the ratio of NAD^+^ to NADH (NAD^+^/NADH) [31]. NAD^+^/NADH is a crucial factor determining metabolic homeostasis in hepatocytes, including fatty acid synthesis, fatty acid oxidation, gluconeogenesis, and glycolysis [32]. In particular, the decrease in NAD^+^/NADH ratio promotes fat accumulation in the liver by reducing fatty acid oxidation and enhancing fatty acid synthesis [21]. Alcohol intake promotes hepatic fat accumulation via various mechanisms, including elevated expression levels of lipogenic genes (e.g., sterol regulatory element-binding protein [SREBP]-1c and its target genes) [33,34,35] and inhibition of genes involved in fatty acid oxidation (e.g., peroxisome proliferator-activated receptor [PPAR]-α target genes) [30,35,36,37]. Notably, CYP2E1-dependent ROS production was shown to inhibit PPAR-α-mediated fatty acid oxidation genes, such as acyl CoA oxidase [30]. Alcohol-induced fat accumulation may, in turn, cause cellular stress and hepatocyte death, which can also be directly stimulated by ethanol and ethanol-derived metabolites [38]. Alcohol-induced hepatocyte injury and inflammation are closely associated with oxidative stress; thus, this section discusses the detailed involvement of oxidative stress in alcohol-induced hepatocyte injury, as well as the role of immune cells in mediating alcohol-induced inflammatory liver injury (Figure 1). In addition, we summarize the messengers linking oxidative stress and inflammation in ALD pathogenesis. Furthermore, we elaborate on experimental ALD models characterized by profound oxidative stress and inflammation and the consequences of modulating oxidative stress and/or inflammation in ALD models.

### 2.1. Alcohol-Induced Hepatocyte Injury

Ethanol is metabolized to acetaldehyde in hepatocytes, mainly via an enzymatic reaction catalyzed by ADHs [39]. There are six closely related ADHs: ADH1A, ADH1B, ADH1C, ADH4, ADH5, and ADH6 [40]. Among these, ADH1A, ADH1B, and ADH1C are responsible for the majority of ethanol oxidation in the liver [41]. Acetaldehyde generated by the enzymatic reaction reacts with DNA and proteins, thereby forming adducts that induce hepatocyte injury. The catalytic cycle of ADH is coupled with the conversion of NAD^+^ to NADH [42]. Aldehyde dehydrogenases (ALDHs) catalyze the conversion of acetaldehyde to acetate using NAD^+^ as a cofactor, which is also converted to NADH [32]. Re-oxidation of NADH to NAD^+^ in the mitochondria has been associated with electron leakage from the mitochondrial respiratory chain and subsequent ROS production [43,44,45]. In addition, ethanol inhibited the expression of antioxidant enzymes (e.g., superoxide dismutase 1) and depleted levels of non-enzyme antioxidants (e.g., glutathione), thereby reducing the cellular ability to modulate oxidative stress [25,26,46,47].

Alternatively, CYP2E1 can be induced by chronic alcohol consumption and can oxidize ethanol to acetaldehyde. CYP2E1 produces ROS, such as O_2_^–^, H_2_O_2_, and ·OH [48,49]. Several animal studies have proposed that CYP2E1 is central to ethanol-induced oxidative stress and hepatic injury. CYP2E1 is mainly located within ER, but also expressed in the mitochondria. The Cederbaum group investigated the role of mitochondrial targeted CYP2E1 in ethanol-induced oxidative stress and mitochondrial damage [50]. Mitochondrial CYP2E1 regulated buthionine sulfoximine-mediated GSH depletion, leading to cell death. Mitochondrial CYP2E1 also contributes to increased levels of ROS and mitochondrial 3-nitrotyrosine and 4-hydroxynonenal protein adducts as well as decreased mitochondrial aconitase activity and mitochondrial membrane potential [50]. Chronic alcohol consumption induced mitochondrial CYP2E1, which plays an important role in ALD. Pharmacological inhibition of CYP2E1 by chlormethiazole reduced liver injury induced by two months of ethanol feeding in rats [51]. Furthermore, chlormethiazole suppressed the development of hepatocellular carcinoma in rats induced by treatment with ethanol and diethylnitrosamine [52]. Lu et al. demonstrated that genetic ablation of the Cyp2e1 gene in mice reduced oxidative stress and prevented ethanol-induced liver injury [30]. In addition, chlormethiazole treatment reduced oxidative stress induced by two-week ethanol feeding in mice [30]. Diesinger et al. reported that novel chimeric inhibitors of CYP2E1 restored the redox balance and rescued liver injury in alcohol-exposed rats [53].

NADPH oxidase (NOX) is an important source of ROS generation which produces superoxide from oxygen using NAD(P)H [54]. NOX1 and NOX4 are abundantly expressed in the liver and hepatocytes [55]. Chronic alcohol consumption increased NOX4 expression in mitochondrial fraction. GKT137831, a NOX4 inhibitor, partially reversed alcohol-induced liver injury, the levels of mitochondrial ROS, mitochondrial DNA, respiratory chain complex IV, and hepatic ATP. Knockdown of NOX4 increased mitochondrial membrane potential and decreased mitochondrial superoxide levels, the number of apoptotic cells, and lipid accumulation [54].Diverse types of cell death, including apoptosis, necroptosis, pyroptosis, and ferroptosis mediate alcohol-induced hepatocyte death [56]. Mitochondria have been highlighted as important locations for ROS-associated cell death [57]. ROS production and oxidative stress caused by ethanol or acetaldehyde reportedly alter the mitochondrial membrane permeability and transition potential [58,59]. This promotes the release of cytochrome c and other pro-apoptotic factors, thereby stimulating the intrinsic pathway of apoptosis [60]. Apoptotic factors released into the cytosol interact with Apaf-1 and caspase-9 to form the apoptosome [61,62,63]. Mitochondrial permeability transition was found to activate caspase-3 in hepatocytes dependent on p38 mitogen-activated protein kinase (MAPK) [64].

Iron overload has been observed in approximately 50% of patients with ALD [65]. Alcohol consumption can decrease the expression of hepcidin through suppression of the transcriptional activity of CCAAT/enhancer binding protein alpha [66]. Hepcidin promotes the degradation of ferroportin, thereby reducing duodenal iron absorption [67]. Downregulation of hepcidin enhances the expression of ferroportin and divalent metal transporter 1 in the duodenum [68]. This is in line with the observation that alcohol intake elevates serum iron levels, serum ferritin levels, and transferrin-iron saturation [69]. In addition to the serum iron levels, hepatic iron is reportedly increased in ALD patients, which may contribute to ROS-associated alcohol toxicity, as iron induces oxidative stress through Fenton reactions [70,71]. Iron overload can also cause cellular damage and death through the process called ferroptosis, a type of iron-dependent programmed cell death [72,73]. There are several crucial regulators of ferroptosis, including lipid peroxidation and iron accumulation [74]. Iron accumulation in cells causes lipid peroxidation and subsequent damage and rupture of the cell membrane, thereby promoting the release of damage-associated molecular patterns (DAMPs) [75]. Iron is believed to play a role in ROS production through several mechanisms, such as iron-containing enzymes (e.g., lipoxygenase) and the Fenton reaction that requires iron [76,77]. In the liver, ferroptosis generates ROS and depletes glutathione levels [78,79]. Ferroptosis has gained momentum as a type of cell death that exacerbates ALD, as evidenced by iron overload observed in the liver of patients with alcohol-related cirrhosis [80]. Moreover, alcohol administration was shown to induce excessive iron accumulation and ferroptosis in animal models [81,82].

ROS are highly reactive and can react with various biological materials ranging from lipids to nucleic acids and proteins. Lipid species reacting with ROS undergo lipid peroxidation and produce 4-hydroxynonenal and malondialdehyde, which can induce several forms of cell death, including apoptosis and ferroptosis [83,84]. Lipid peroxidation products can also bind to DNA and enhance carcinogenesis by producing etheno-DNA adducts [85,86]. Proteins that react with ROS modify their structures and functions, possibly resulting in neoantigens that can induce an immune response [87].

Building on the concept that oxidative stress is involved in hepatocyte injury in ALD, several recent reports have investigated the therapeutic potential of suppressing oxidative stress-associated signaling pathways. For example, Ma et al. demonstrated that inhibition of ASK1 and p38MAPK, which relay oxidative stress to cell death signaling, afforded protection against hepatocyte death induced by ethanol feeding in mice [88]. In addition, recent studies have demonstrated that the Nrf2/ARE pathway might be a useful target for reducing ethanol-induced oxidative stress and liver injury [20,89,90,91,92,93,94].

### 2.2. Immune Cells Mediating the Crosstalk between Oxidative Stress and Inflammation in ALD

Alcohol-exposed hepatocytes that undergo oxidative stress-induced cellular injury and death produce a variety of inflammatory mediators, such as cytokines, chemokines, and DAMPs (e.g., high-mobility group box 1 protein and mitochondrial DNA), which can, in turn, activate immune reactions and inflammation [95,96,97,98]. DAMPs are recognized by Toll-like receptors (TLRs) and NOD-like receptors, such as NLRPs, which are expressed in hepatocytes and immune cells [99,100]. DAMP-mediated activation of these receptors intensifies innate immunity-related inflammatory pathways in ALD, along with enhanced expression of cytokines, chemokines, and adhesion molecules that promote the infiltration and/or activation of innate immune cells, such as neutrophils, macrophages, and Kupffer cells [101,102,103]. In addition, alcohol consumption augments ROS levels and lipid peroxidation, facilitating the production of protein adducts with malondialdehyde and 4-hydroxynonenal, which may function as neoantigens and activate adaptive immunity mediated by T and B cells [104].

As stated above, hepatic inflammation during ALD progression is associated with the infiltration and activation of inflammatory cells, such as macrophages and neutrophils, whose actions are associated with ROS production [105,106]. Oxidative stress and inflammatory cell activation often mutually affect each other; ROS derived from damaged cells activate inflammatory cells, and the activation of these immune cells further enhances oxidative stress by producing ROS and reactive nitrogen species such as peroxynitrite and nitric oxide [107,108]. This section highlights the detailed roles of oxidative immune cells in the progression of ALD.

#### 2.2.1. Neutrophils

**Figure 2:**
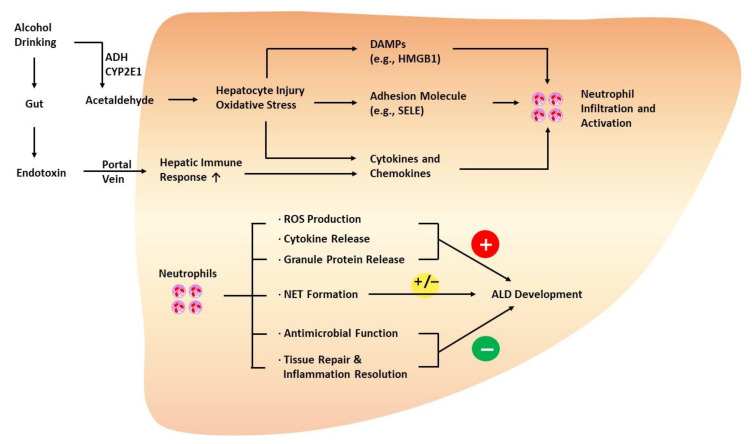
Role of neutrophils in the development of ALD. Injured hepatocytes with oxidative stress promote neutrophil infiltration and activation via the release of DAMPs, cytokines, and chemokines. In addition, endothelial cells upregulate adhesion molecules, such as SELE, to facilitate hepatic neutrophil infiltration. Neutrophils play both protective and detrimental roles during ALD progression. Generally, neutrophils are known to exacerbate ALD via oxidative burst, ROS production, cytokine release, and the release of granule proteins (e.g., myeloperoxidase). However, neutrophils also express antimicrobial factors, such as lipocalin 2, and play a crucial role in affording protection against infection in patients with ALD. Neutrophils are also involved in tissue repair by releasing HGF and inflammation resolution, delaying the progression of ALD. NETs not only augment hepatocyte injury but also mediate the antimicrobial function of neutrophils. HGF, hepatocyte growth factor; HMGB1, high-mobility group box 1 protein; NET, neutrophil extracellular trap; SELE, E-selectin.

Neutrophils are the most abundant subset of leukocytes in the circulation and participate in various processes of immune reactions and inflammation [109]. For example, in response to oxidative hepatic injury during ALD progression, neutrophils migrate from the circulation to the affected tissue, regulated by chemokines, cytokines, and adhesion molecules that attract and activate neutrophils in an orchestrated manner (Figure 2) [110,111,112].

Hepatic neutrophil infiltration is enhanced after chronic alcohol consumption and acute and heavy alcohol exposure [113,114,115,116]. In particular, binge ethanol intake can promote hepatic neutrophil infiltration and elevate circulating neutrophils in alcoholic individuals [117], which is postulated to contribute to the switching of chronic ASH with macrophage inflammation to AH with neutrophil infiltration [118]. Animal models that mimic the acute-on-chronic alcohol consumption pattern of alcoholics have also been reported to exhibit marked neutrophil infiltration in the liver. The National Institute on Alcohol Abuse and Alcoholism (NIAAA) model is characterized by a combination of 10 days of *ad libitum* feeding on the Lieber–DeCarli ethanol diet and a single binge ethanol feeding (chronic-plus-binge ethanol feeding), recapitulating the features of early-stage AH [119]. In the livers of mice subjected to the NIAAA model, neutrophil-recruiting chemokines, such as CXCL1 and interleukin (IL)-8, were upregulated, along with substantial neutrophil infiltration, similar to the liver of patients with ALD [115].

While oxidative stress-associated hepatocyte damage and death promote neutrophil activation and recruitment to the site of injury, activated neutrophils can also produce ROS through oxidative burst, which is one of the mechanisms underlying neutrophil functions [105,120]. Other mechanisms include phagocytosis, degranulation, the release of proteases (e.g., neutrophil elastase), and neutrophil extracellular trap formation [121]. Oxidative burst is mediated by NOX2 and its association with components of the NOX2 complex, such as p47^phox^, p67^phox^, p40^phox^, and p22^phox^ [122,123]. Neutrophilic ROS production via oxidative bursts may further stimulate hepatocyte injury [117,124,125].

Li et al. investigated the critical role of the neutrophilic IL-6-p47^phox^-oxidative stress pathway in the development of ALD [117]. Mice deficient in the gene encoding microRNA-223 (miR-223) were more susceptible to hepatic neutrophil infiltration and neutrophil ROS production when subjected to the chronic-plus-binge ethanol feeding model of ALD [117]. Mechanistically, the authors showed that miR-223 inhibited the IL-6-p47^phox^-ROS pathway in neutrophils, thereby decreasing the severity of the alcohol-induced liver injury. In addition, the authors documented numerous circulating neutrophils and higher levels of serum alanine aminotransferase (ALT) and aspartate aminotransferase (AST) in alcoholics with recent excessive drinking than in healthy individuals. Roh et al. demonstrated that the upregulation of CXCL1 and subsequent neutrophil infiltration in mice subjected to chronic-plus-binge ethanol feeding depended on TLR2 and TLR9 signaling [126].

IL-17 is reportedly elevated in patients with AH and can affect the function of neutrophil-attracting chemokines [127]. Ma et al. reported that deletion of the gene encoding IL-17RA reduced the expression level of CXCL1 and delayed the development of alcohol-associated liver cancer, indicating that IL-17 signaling promotes hepatocellular carcinoma in ALD [128].

Typically, neutrophils have been recognized as a deleterious cell type that exacerbates alcohol-induced liver injury and inflammation; however, studies have also revealed the potential benefits of neutrophils. Neutrophil dysfunction predicts the poor prognosis of AH with cirrhosis, which has been attributed to uncontrolled infection [129]. Neutrophils may participate in tissue repair and inflammation resolution to maintain tissue homeostasis. For instance, neutrophil-mediated ROS production stimulates the conversion of proinflammatory macrophages (Ly6C^hi^CX_3_CR_1_^lo^) to pro-resolving macrophages (Ly6C^lo^CX_3_CR1^hi^) during acute liver injury [130,131]. Further studies are warranted to elucidate the complex functions of neutrophils. The advent of single-cell analysis may accelerate the identification of a distinct subset of neutrophils that differentially participate in the pathogenesis of ALD.

#### 2.2.2. Macrophages

During the course of ALD progression, the sustained inflammatory environment leads to hepatic monocyte infiltration. Monocytes infiltrating the liver differentiate into macrophages [132]. Several subpopulations of macrophages exist in the liver, including resident macrophages, Kupffer cells, and monocyte-derived macrophages [133]. The population of macrophages was shown to be elevated in the liver of patients with ALD, as well as in experimental ALD models [134,135]. In addition, a study conducted in rats reported that depletion of hepatic macrophages by gadolinium chloride treatment reduced alcohol-induced hepatic inflammation [136], indicating the importance of hepatic macrophages in the development of ALD.

Alcohol consumed is absorbed through the gastrointestinal tract; thus, the gut is one of the first organs whose integrity is altered by alcohol intake [137,138,139,140].

Ethanol and ethanol metabolites modulate the physiology of the intestine through several mechanisms. First, ethanol and ethanol metabolites may directly damage the intestine epithelial cells. In humans, ethanol consumption results in acute subepithelial bleb formation and hemorrhagic erosions [141]. Chronic alcohol consumption alters the histological properties of the duodenal mucosa (e.g., decreased surface area) [142]. In rats, hemorrhagic erosions of the proximal small intestine with epithelial cell loss were observed upon acute administration of ethanol [143]. In mice, submucosal blebbing and ulceration of villi in the ileal small intestine were observed upon acute ethanol exposure [144]. A study using Caco-2 monolayers demonstrated that ethanol treatment induced apoptosis, which was augmented by exposure to *E. coli* [145,146]. Oxidative stress-associated mitochondrial dysfunction has been suggested as a potential mechanism underlying the damage of intestinal epithelial cells by ethanol metabolites such as fatty acyl ethyl esters [147].

Secondly, ethanol and ethanol metabolites impair the integrity of tight junctions in epithelial barriers, and the interaction between zonula occludens-1 and occludin is a hallmark of tight junction formation [148]. Ethanol and acetaldehyde cause redistribution of occludin from the intestine epithelial tight junctions [149,150,151,152]. Oxidative stress has also been suggested as a crucial mediator of alcohol-associated alteration of tight junctions. A study using Caco-2 cells revealed that ethanol treatment disrupted barrier function and damaged microtubules through inducible nitric oxide synthase (iNOS)-dependent ROS production [153]. The iNOS-dependent ROS production was found to be the mechanism by which ethanol gavage stimulates the intestinal permeability in rats [154].

Lastly, alcohol consumption can change the composition and the number of microbiota in the intestine, which may lead to an increase in gut permeability [155]. For example, patients with ALD have a lower population of *Faecalibacterium prausnitzii*, which produce butyric acid [156,157]. Butyric acid contributes to the intestine epithelial barrier by maintaining the expression of the tight junction proteins and mucins [158,159]. *Bacteroidetes* are reportedly decreased in the individuals with excessive alcohol consumption, whereas *Proteobacteria* are increased in individuals with chronic drinking [160]. Bacterial overgrowth has been also observed in experimental ALD models and patients with ALD. For instance, three-week feeding of ethanol increased the population of bacteria in the small intestine of mice [161]. Bacterial growth is reportedly profound in humans with chronic alcohol abuse [162,163].

Alcohol-induced dysregulation of the intestinal barrier mediated by the mechanisms above is postulated to increase gut permeability to Gram-negative bacterial endotoxin, promoting the transfer of endotoxin to the circulation and eventually to the liver via the portal vein [164,165,166,167]. Pathogen-associated molecular patterns (PAMPs) such as lipopolysaccharide (LPS) associated with the incoming bacteria interact with TLR4 in macrophages, including Kupffer cells, stimulating the production and release of inflammatory cytokines and chemokines that further augment inflammation and recruit monocytes [111,168]. Apart from PAMPs, DAMPs may also activate Kupffer cells in the context of sterile inflammation during ALD development, which, in turn, stimulates the release of inflammatory mediators that promote the infiltration and activation of monocytes/macrophages [95,169,170]. One possible mechanism is dependent on the action of inflammasomes, known to activate caspase-1 and secrete inflammatory mediators, including IL-1β and IL-18 [171,172].

There are two distinct types of infiltrating monocytes depending on Ly6C expression levels. Ly6C^hi^ monocytes are proinflammatory and tissue-damaging, whereas Ly6C^lo^ monocytes mediate patrolling, anti-inflammatory, and tissue-reparative functions [173]. The number of Ly6C^hi^ monocytes was found to be increased in experimental ALD [135]. Ly6C^hi^ cells participate in the efferocytosis of apoptotic hepatocytes, which is the process through which dying cells are removed by phagocytic cells such as macrophages [174]. Accordingly, Ly6C^hi^ cells may switch to Ly6C^lo^ cells after efferocytosis of hepatocytes [135,175].

The production of oxidants in activated macrophages primarily occurs through the action of NOX [123,176]. Chronic ethanol feeding-induced ROS production in Kupffer cells is dependent on the action of NOX and p47^phox^ [177]. NOX-derived ROS are key players mediating nuclear factor-kappa B (NF-κB) activation and subsequent production of tumor necrosis factor (TNF)-α in Kupffer cells upon ethanol administration [177], thus indicating that oxidative stress may enhance the inflammatory function of Kupffer cells and contribute to ALD pathogenesis. Furthermore, ROS can sensitize Kupffer cells to LPS. In animals subjected to chronic ethanol feeding, LPS-induced ROS production was enhanced in Kupffer cells, which was attenuated by inhibiting NADPH oxidase [178]. LPS sensitization in Kupffer cells by NADPH oxidase-derived ROS (e.g., LPS-stimulated TNF-α production) was in part attributed to the activation of extracellular signal-regulated kinase (ERK), a stress kinase activated by ROS [178].

Despite the abundance of the hepatic resident macrophages, as well as a marked increase in the population of hepatic macrophages upon alcohol consumption, there remains a gap in the knowledge regarding the role of macrophages in ALD pathogenesis. Identifying signaling molecules that link oxidative and inflammatory functions of macrophages, as well as those responsible for the interdependence between the polarization status of macrophages and their oxidative ability, will open new avenues for future research.

#### 2.2.3. Other Types of Immune Cells

Neoantigens generated by ROS-induced alteration of protein structures can result in T cell activation [179]. Activated T cells promote the progression of ALD by releasing proinflammatory cytokines such as TNF-α, IL-1, and IL-17 [180]. In addition, the cytotoxic property exerted by CD8^+^ T cells contributes to the progression of ALD [181]. In addition to CD8^+^ T cells, CD4^+^ T cells also contribute to ALD development by releasing multiple types of cytokines. For example, Th1 cells help activate macrophages and exacerbate liver injury and inflammation by releasing cytokines such as interferon (IFN)-γ, IL-2, and TNF-α [182,183]. Th17 cells produce IL-17, which enhances liver injury and inflammation; however, Th17 cells can produce IL-22, which possesses anti-apoptotic and antioxidant properties through STAT3 activation [127,184,185,186].

Natural killer T (NKT) cells are a subset of T cells that express T cell receptors; however, they also express unique marker proteins such as NK1.1, CD161, and CD56 in humans [187]. Although NKT cells are presumed to be involved in accelerating ALD progression by activating hepatic macrophages in rodent models, limited data are available to determine whether NKT cells contribute to ALD progression in humans [180]. Mathews et al. demonstrated that chronic-plus-binge ethanol feeding in mice activated invariant NKT cells, also known as type 1 NKT cells, which release mediators that recruit neutrophils to the liver and promote the development of ALD [114]. In contrast, type 2 NKT cells may inhibit the progression of ALD by suppressing the action of type 1 NKT cells [188].

Mucosa-associated invariant T (MAIT) cells are a subset of innate-like T cells that possess a conserved invariant T cell antigen receptor (TCR) α-chain [189]. The composition of the chain is different between species. For example, humans possess Vα7.2-Jα33, whereas mice possess Vα19-Jα33 [190].

MAIT cells are abundantly observed in the liver of humans [191]. Approximately 30% of intrahepatic T cells are considered MAIT cells in humans; however, mice have markedly lower population of MAIT cells, which makes it difficult to precisely understand the function of MAIT cells [192]. MAIT cells have been demonstrated to inhibit bacterial infection [193]. Mechanistically, invariant TCRs in MAIT cells interact with riboflavin (vitamin B2) derivatives that are presented by the major histocompatibility complex class I-related protein 1 [194]. Mechanisms that are independent of TCRs are also known to mediate the antibacterial function of MAIT cells. For instance, IL-12 and IL-18 may activate MAIT cells, thereby producing numerous types of cytokines, including TNF-α, IFN-γ, and IL-22, and regulating immune responses [194].

Riva et al. reported that patients with alcoholic cirrhosis and severe alcoholic hepatitis have lower levels of MAIT cells in the circulation and weakened antibacterial potency [195]. They also reported that intestinal bacterial antigens and metabolites reduced the production of antibacterial cytokines by MAIT cells in vitro [195]. Alcohol consumption-associated dysfunction of the intestinal epithelial barrier leads to an increased gut permeability which induces the migration of bacterial antigens and metabolites to the portal circulation. These may reduce the number of MAIT cells in the circulation as well as in the liver, which may in part explain the reduced antibacterial capability observed in individuals with chronic alcohol consumption.

### 2.3. The Role of MicroRNAs in the Crosstalk between Oxidative Stress and Inflammation in ALD

MicroRNAs (miRNAs) are key players in ALD. The landscape of miRNA expression is reportedly altered under pathological conditions [196,197,198]. Dysregulated miRNAs contribute to the regulation of pathophysiological pathways in ALD via several different mechanisms (Table 1). miRNAs can directly bind to the 3′UTR of target genes, leading to degradation or translational repression of target mRNAs. In contrast, miRNAs sometimes enhance translational activation [199]. Furthermore, miRNAs not only mediate gene regulation, but several miRNAs possessing a GC-rich motif (e.g., let-7b, miR-21, and miR-29a) can serve as ligands for TLRs [200]. Herein, we discuss the role of miRNAs in inflammation, cell death, and oxidative stress during ALD and their regulatory mechanisms.

The most overexpressed miRNA in the liver tissue of patients with AH when compared with normal livers is miR-182 [197]. Increased miR-182 levels are associated with disease severity. miR-182 is mainly found in the ductular reaction cells. In cholangiocytes, miR-182 reportedly targets SLC1A1 and CFL1, whereas miR-182 increases the levels of proinflammatory genes such as CCL20, CXCL1, and IL-8. In addition, miR-182 enhanced IL-6 mRNA levels in hepatocytes and macrophages. Blocking miR-182 using a decoy inhibited liver injury, bile acid accumulation, and proinflammatory genes [197]. Circulating miR-155 and miR-155 levels in hepatocytes and macrophages were elevated in ALD [203,204]. miR-155 induced M1 macrophage polarization by targeting Cebpb and promoted TNF-α production in macrophages [205]. miR-155 knockout mice were found to be resistant to alcohol-induced fatty liver and inflammation [206]. Let-7, a TLR7 ligand, contributes to the hepatic inflammatory response in AH [201]. Ethanol was shown to stimulate the release of let-7b in microvesicles originating from hepatocytes. Hepatic expression levels of let-7b positively correlated with IL-8 and nuclear enriched abundant transcript 1 (NEAT1) expression levels in patients with AH. Activation of TLR7 may contribute to the induction of a subset of inflammatory genes, such as IL-8 and TNF-α [201]. Therefore, miRNAs appear to play a role in the regulation of the inflammatory response associated with ALD.

In addition, miRNAs mediate hepatocyte death in alcohol-associated hepatitis. Elevated IL-1 levels were detected in patients with AH [213]. NLRP3 inflammasome activation and caspase-1-mediated pyroptosis in hepatocytes are reportedly enhanced during ALD [10]. Pyroptosis is regulated by miR-148a, a miRNA abundant in the liver. The miR-148a expression level was greatly decreased in patients with AH and in ALD animal models. Decreased miR-148a expression level by ethanol was found to be responsible for thioredoxin-interacting protein (TXNIP) overexpression. TXNIP-dependent inflammasome activation contributes to hepatocyte pyroptosis. Moreover, miR-148a non-canonically increased the mRNA stability of ADH4 and CYP2B6 by directly binding to the coding sequence and 3′UTR sequence, respectively [210,211]. Caspase-3-mediated apoptosis was shown to be regulated by miRNA(s) in alcohol-associated hepatitis. Fan et al. identified a miRNA-E3 ubiquitin ligase regulatory network for hepatocyte death pathways [202]. miR-150-5p negatively regulated the E3 ligase cytokine-inducible SH2 containing protein (CISH). As Fas-associated protein with death domain, (FADD) is a CISH substrate, ubiquitination of FADD was reduced in the NIAAA model of ethanol-induced liver injury, thus resulting in an increased extent of caspase-3 activation and programmed cell death [202]. These results suggest that miRNAs play an important role in diverse types of hepatocyte death, including pyroptosis and apoptosis.

Additional evidence suggests that oxidative stress-induced miRNA may contribute to the pathology of ALD. Ethanol feeding reduced levels of augmenter of liver regeneration (ALR). ALR deficiency-mediated oxidative stress increased miR-540, which disturbed peroxisomal and mitochondrial lipid homeostasis [209].

miRNAs also play an important role in alcohol-associated oxidative stress. Ethanol can induce miR-214 expression in liver cells [208]. miR-214 was found to directly bind to the 3′UTR of glutathione reductase (*GSR*) and cytochrome P450 oxidoreductase (*POR*) genes. Reduced *GSR* and *POR* levels induced by miR-214 promoted ethanol-induced oxidative stress. In a rat model of alcoholic fatty liver diseases, miR-181b-5p levels were elevated [207]. Inhibition of miR-181b-5p attenuated oxidative stress. Silencing miR-181b-5p increased protein inhibitors of activated STAT1 to suppress oxidative stress and inflammatory response [207]. miR-241 and miR-181b-5p increased by ethanol may induce oxidative stress.

In contrast, the miR-223 level increases in serum and neutrophils in chronic-plus-binge ethanol feeding, and miR-223 attenuates the IL-6-p47^phox^-oxidative stress pathway in neutrophils [117]. Therefore, miR-223 inhibits neutrophil infiltration and protects against alcohol-induced liver injury. Interestingly, the neutrophilic miR-223 expression level was lower in aged mice than in young mice [214]. Aging stimulates the susceptibility to acute and chronic alcohol-induced liver injury by inhibiting the neutrophilic SIRT1-C/EBPα-miR-223 axis. miR-219a-5p attenuated p66shc-mediated ROS in ALD [212]. Protocatechuic acid, a component of green tea, can induce miR-219a-5p expression, thereby ameliorating ALD by reducing ROS formation. These findings suggest that miRNA modulators could play a protective role in ALD by controlling the oxidation pathway. Collectively, miRNAs are major contributors to oxidative stress and inflammatory liver injury in ALD.

## 3. Therapeutic Strategies Targeting Oxidative Stress and Inflammation

### 3.1. Current Therapies for Severe AH

Corticosteroids, such as prednisolone, are recommended as first-line therapy for patients with severe AH. Corticosteroids can reduce short-term mortality within 28 days in patients with severe AH [215]. However, a long-term follow-up study revealed the absence of any survival benefits in patients treated with corticosteroids when compared with controls [216].

Pentoxifylline is the second-line therapy employed in corticosteroid non-responders and patients with corticosteroid contraindications. It is a phosphodiesterase inhibitor that suppresses TNF-α and leukotriene synthesis. As TNF levels are reportedly elevated in the sera of patients with acute and chronic AH and an increase in TNF levels during the hospital course is related to patient mortality, treatment with pentoxifylline was shown to improve short-term survival in patients with severe acute AH [213,217,218]. In particular, pentoxifylline decreased the likelihood of patients developing hepatorenal syndrome [217]. In addition, pentoxifylline can reduce inflammation and exhibits antioxidant properties [219]. Furthermore, it can inhibit xanthine oxidase. Therefore, pentoxifylline can reduce superoxide and hydroxyl radicals. However, another clinical trial (STOPAH, steroids, or pentoxifylline for alcoholic hepatitis) concluded that pentoxifylline did not affect patient survival [220].

### 3.2. Antioxidant Therapy

N-acetylcysteine (NAC), a glutathione precursor, is a well-known antioxidant. NAC has been used as an antidote for acetaminophen-induced liver toxicity [221]. Given that NAC possesses anti-inflammatory and antioxidant properties, it has been suggested as a treatment for ALD [222]. In a study by Badger et al., ethanol was administered to Sprague-Dawley rats by an intragastric cannula and infused with liquid diets using total enteral nutrition [223]. NAC treatment enhanced the cytosolic antioxidant capacity and inhibited ethanol-induced lipid peroxidation. In addition, NAC treatment ameliorated ethanol-induced liver injury and inflammation and maintained the glutathione content [223]. NAC treatment was evaluated in an acute ethanol-induced liver damage mouse model [224]. Pretreatment with NAC prior to a single dose of ethanol prevented acute ethanol-induced lipid peroxidation and glutathione depletion, as well as reduced TNF-α mRNA expression level. Interestingly, NAC administration after ethanol treatment exacerbated acute ethanol-induced liver injury and lipid peroxidation. Therefore, NAC plays a dual role in acute ethanol-induced liver injury, depending on the time of administration. A randomized clinical trial assessing NAC treatment alone or in combination with corticosteroids was performed to evaluate whether antioxidant therapy can improve survival in patients with acute AH [225]. NAC treatment alone or in combination with corticosteroids failed to improve 6-month survival in patients with severe AH. Similarly, another randomized multicenter controlled trial for enteral nutrition with or without NAC for treating severe acute AH failed to show survival benefits [226]. The AAH-NAC study group revealed that prednisolone plus NAC increased one-month survival when compared with the prednisolone-only group; however, the three-month or six-month mortality did not differ significantly between the prednisolone plus NAC and prednisolone-only groups [227]. The six-month mortality attributed to hepatorenal syndrome and infections was less frequent in the prednisolone plus NAC group than in the prednisolone-only group. A retrospective analysis also demonstrated that a combination of prednisolone and NAC afforded no survival advantages over prednisolone alone in severe AH [228].

S-adenosyl-L-methionine (SAMe) is a methyl donor that regulates GSH synthesis. A randomized, placebo-controlled, double-blind, multicenter clinical trial suggested that long-term treatment with SAMe reduced overall mortality and delayed liver transplantation in patients with alcoholic liver cirrhosis, especially in Child class A or B [229]. However, another clinical trial indicated that a 24-week SAMe therapy did not improve clinical or biochemical parameters in ALD [230].

Metadoxine, another antioxidant, is an ionic complex of the pyridoxine-pyrrolidone molecule [231,232]. The beneficial effects of metadoxine in ALD have been reported. Metadoxine reportedly prevents redox imbalance in hepatocytes and inhibits TNF-α secretion in hepatic stellate cells caused by ethanol or acetaldehyde [233]. In addition, metadoxine improved liver function and stimulated fatty liver recovery [234]. Metadoxine plus glucocorticoids significantly improved short-term survival rates in patients with severe AH and inhibited encephalopathy and hepatorenal syndrome [235]. Metadoxine plus pentoxifylline also improved the 3- and 6-month survival rates in patients with severe AH when compared with pentoxifylline alone [236]. Therefore, metadoxine treatment in combination with current therapies should be considered.

### 3.3. IL-1 Inhibitors

IL-1β is a proinflammatory cytokine that acts by engaging the type I IL-1 receptor. IL-1β levels are elevated in ALD [237]. Activation of pattern recognition receptors induces IL-1β gene expression. Pro-IL-1β is cleaved into mature IL-1β via the inflammasome complex [171]. Gasdermin D membrane pores are required for IL-1β release [238]. Caspase-1 inflammasome activation and IL-1 signaling promote the pathogenesis of alcohol-induced inflammation, steatosis, liver damage, and fibrosis [172]. Therefore, pharmacological inhibition of IL-1Ra has been suggested as an attractive therapeutic intervention. Anakinra, an IL-1 receptor antagonist, is an FDA-approved drug for rheumatoid arthritis, Still’s disease, familial cold auto-inflammatory, and Muckle-Wells syndrome [239]. Treatment with anakinra ameliorated ALD development in vivo [172]. A combination of drugs, including anakinra, was evaluated in patients with alcohol-associated hepatitis. In the Phase IIB clinical trial (the DASH study), a combination of anakinra, pentoxifylline, and zinc sulfate was evaluated to improve clinical outcomes in patients with severe AH when compared with methylprednisolone, an accepted standard therapy [239]. The DASH study has been completed, and a Phase 2 trial of anakinra (plus zinc) or prednisone in patients with severe AH remains ongoing (NCT01809132). These studies will determine the clinical efficacy and safety of anakinra when compared with standard corticosteroid treatment in patients with severe AH.

Canakinumab is a monoclonal antibody inhibitor of IL-1, developed by Novartis [240]. This drug is approved for cryopyrin-associated periodic syndromes, rare and serious auto-inflammatory diseases, and active Still’s disease. A Phase 2 clinical trial of IL-1 signal inhibition in AH (ISAIAH) will assess the histological improvement in AH after 28 days of canakinumab treatment and the potential benefits of the IL-1β antibody (NCT03775109). Collectively, the inhibition of IL-1 signaling by IL-1Ra or anti-IL-1 antibodies is an attractive drug target for ALD.

### 3.4. IL-22

IL-22 is a pluripotent T cell-derived cytokine with antioxidant, anti-apoptotic, anti-steatotic, antimicrobial, pro-regenerative, and anti-fibrotic properties [241]. IL-22 mainly induces STAT3 activation by binding to the heterodimeric IL-22R1 and IL-10R2 receptors, contributing to the upregulation of anti-apoptotic and mitogenic genes [242]. IL-22 treatment attenuated ethanol-induced liver injury via STAT3 activation [243]. F-652 is a recombinant fusion protein containing two human IL-22 molecules linked to human immunoglobulin G2-Fc. Intravenous administration of F-652 to healthy subjects is reportedly safe and well-tolerated [244]. The safety and efficacy of F-652 were evaluated in a Phase 2 dose-escalating study [245], with up to 45 μg/kg of F-652 found to be safe. In addition, administration of F-652 improved the Lille score and model for end-stage liver disease (MELD) score, downregulated inflammatory cytokine markers, and upregulated regeneration markers [245]. These results suggest that IL-22 may have therapeutic potential in treating ALD [246].

### 3.5. Anti-TNFα Antibody, Infliximab

The proinflammatory cytokine TNFα plays an important role in the pathophysiology of ALD. It mediates portal and systemic haemodynamic derangements in alcoholic hepatitis [247]. Infliximab, a monoclonal anti-TNFα antibody, is used in the treatment of various inflammatory diseases, such as rheumatoid arthritis, Crohn’s disease, and ankylosing spondylitis. The safety, tolerance and clinical effects of infliximab has been evaluated in severe AH. First, a randomized controlled pilot study showed that infliximab was well tolerated and Maddrey’s score significantly improved in patients with severe AH who received a combination of steroids with infliximab at day 28 [248]. Another clinical trial evaluated a single infusion of infliximab on severe AH patients. This study suggests that infliximab treatment improved serum bilirubin levels, the Maddrey score, the neutrophil count and C-reactive protein levels [249]. Unexpectedly, a double-blind randomized controlled trial showed that three infusion of 10 mg/kg of infliximab in combination with prednisolone caused high probability of death within two months due to the high prevalence of severe infections [250]. The Sarin group also concluded that patients with severe AH who received a single dose of infliximab showed the improvement in parameters of disease severity and patient survival, but also a risk of developing serious infections such as pneumonia and pulmonary tuberculosis [251].

### 3.6. Obeticholic Acid

The bile acid receptor farnesoid X receptor (FXR) is a nuclear receptor, which is highly expressed in the liver and intestine. FXR has important roles in regulation of lipid absorption, glucose metabolism as well as the maintenance of bile acid homeostasis [252,253,254]. Bile acid-FXR-FGF15 signaling regulates hepatic Cyp7a1 and lipid metabolism [255]. In addition, FXR attenuates liver inflammation [256]. In an experimental mouse model of ALD, a FXR activator, WAY-362450, decreased alcohol-induced CYP2E1 and ameliorated oxidative stress in liver [257]. FXR knockout mice were more susceptible to alcohol-induced liver injury due to impaired FoxO3a-mediated autophagy [258]. A selective FXR agonist, obeticholic acid (Ocaliva, Intercept Pharmaceuticals) is approved for the treatment of primary biliary cholangitis [259]. A double-blind, placebo-controlled phase 2 clinical trial of obeticholic acid in patients with moderately severe AH was completed (NCT02039219). According to the results of a phase 3 clinical trials of obeticholic acid for non-cirrhotic, non-alcoholic steatohepatitis (NASH) (the FLINT study), patients treated with obeticholic acid experienced severe pruritus. Moreover, obeticholic acid treatment caused the elevation of total serum cholesterol and LDL cholesterol and a decreased in HDL cholesterol [260]. Recently, FDA restricts use of obeticholic acid in primary biliary cholangitis patients with cirrhosis due to risk of serious liver injury. Therefore, the use of obeticholic acid to treat ALD should be carefully evaluated. The Schnabl group showed that the intestine-restricted FXR agonist fexaramine protected mice from ethanol-induced liver injury and that FGF19 treatment similarly has a beneficial effect on alcoholic steatohepatitis [255]. These strategies can be considered to reduce unfavorable effects of systemic FXR agonists [256].

## 4. Conclusions and Perspectives

Although the involvement of oxidative stress in the pathogenesis of ALD has been previously established, detailed mechanisms underlying the relationship between oxidative stress and diverse pathogenic players of ALD continue to be elucidated, given the expansion in our knowledge regarding cell death, immune reactions, and inflammation in the context of ALD. Accumulation of the clinically relevant knowledge regarding the role of oxidative stress and inflammation will help develop optimal experimental ALD models that will facilitate rapid screening of and pharmacological studies on potential therapeutic agents. Although no approved medications for ALD have been developed based on a strategy specifically targeting oxidative stress, recent clinical trials suggest that antioxidant drugs or drugs inhibiting inflammatory liver injury may be used to treat patients with ALD in the future.

## Figures and Tables

**Table 1:** Aberrant microRNA expression in ALD and the associated pathological effects.

microRNA	Status in ALD	Targets	Effects	References
Let-7b	Up	TLR7 activation	↑hepatic inflammatory response	[201]
miR-150-5p	Up	CISH	↑FADD-mediated programmed cell death	[202]
miR-155	Up	Cebpb	↑M1 macrophage polarization ↑fatty liver	[203,204,205,206]
miR-181b	Up	PIAS1	oxidative stress and inflammation	[197,207]
miR-182	Up	SLC1A1 CFL1	↑liver injury and inflammation	[197]
miR-214	Up	GSR POR	↑oxidative stress	[208]
miR-223	Up	IL-6	↓oxidative stress	[117]
miR-540	Up	PPARα, PMP70, ACOX1, CPT1a	↑hepatic steatosis	[209]
miR-148a	Down	TXNIP	↑TXNIP-dependent inflammasome activation ↑ADH4 and CYP2B6	[10,210,211]
miR-219a-5p	Down	P66shc	↑oxidative stress	[212]

↑: increased, ↓: decreased.
